# Repeated *Plasmodium vivax* malaria relapses in a Peruvian sailor

**DOI:** 10.1186/s12936-015-0959-x

**Published:** 2015-12-01

**Authors:** Adam P. McFarland, Juan F. Sanchez, Alejandro Mercado, Julio A. Ventocilla, Sofia Cavalcanti, Sofia Gonzalez, Andres G. Lescano

**Affiliations:** School of Medicine and Health Sciences, The George Washington University, Washington, DC USA; Department of Parasitology, U.S. Naval Medical Research Unit No. 6 (NAMRU-6), Av. Venezuela Cdra. 36 S/N. Bellavista, Callao, 03 Peru; Centro Medico Naval “Cirujano Mayor Santiago Tavara”, Peruvian Navy, Callao, Peru; School of Public Health and Management, Universidad Peruana Cayetano Heredia (UPCH), Lima, Peru

## Abstract

Two *Plasmodium vivax* recurrences in a Peruvian sailor with weight above the 60 kg (cap for primaquine dosage) highlight the importance of adequate radical cure weight dosage for patient treatment and control efforts, particularly within the military.

## Case report

A 38 year old, 76 kg, 1.68 m male Peruvian sailor presented to emergency room of the Peruvian Navy hospital in Lima on February 15, 2011. The patient reported a seven day history of fever, chills, and headache without significant past medical history. He had been deployed to a military base in a malaria-endemic area called VRAEM (Valle del Río Apurímac, Ene y Mantaro) from March 2010 to January 2011. The patient did not receive any pre-deployment prophylactic anti-malarials. His physical examination showed 39 °C fever, but had no neurological or respiratory symptoms, severe malaria, jaundice, nor liver or kidney infection or failure. His platelet levels were 120,000/mm^3^ and leukocytes at 3400 cells/µL; all other laboratory results were in normal ranges. The patient was diagnosed with *Plasmodium vivax* (*P. vivax*) malaria confirmed by blood smear on February 16th, and received a regimen of 3 days of chloroquine concurrent with 7 days of primaquine. He received 1 g of chloroquine on the first and second day of treatment, and 500 mg on the third day. He received 30 mg of primaquine daily for the entire 7 days. The drug administration was in accordance with the Peruvian Ministry of Health standards. Treatment was provided under supervision by health professionals with complete adherence. Fever resolved by the fourth day of hospitalization. The final thick smear on day 7 was negative. The patient was discharged with no symptoms on March 18, 2011.

On May 20, 2011, the patient presented again to the emergency room with a 1-week history of similar symptoms. He reported no travel to other endemic areas since the previous hospitalization. He weighed 75 kg, had 39 °C fever, his platelet level was 202,000/mm^3^, and leukocytes at 4300 cells/µL. The patient had antibodies to the hepatitis B core antigen, but was negative for the hepatitis B surface antigen and other infectious diseases and parasites. A thick smear performed on May 20th tested positive again for *P. vivax*. The patient was provided the same treatment and dose as the initial (February) episode. His fever had resolved by the fourth day of hospitalization. The final thick smear was negative.

The patient returned a third time with similar symptoms in August, 2011. He reported no travel to a malaria endemic area since his previous hospitalization in May except to visit family in Pucallpa city, Peru (Ucayali department, 52 cases of *P. vivax* in 2011), during which he only stayed in the urban area where no malaria transmission occurs, suggesting a low chance of re-infection. A thick smear tested positive for *P. vivax*. The patient was prescribed primaquine for 14 days at 30 mg per day and was released from the hospital after fully completing treatment under the supervision of Navy health staff. Microsatellite analysis was performed using the initial (February) patient blood sample and the second recurrence (August) thick smear in order to determine the genetic relationship between episodes. The six microsatellite markers tested were identical between the two samples (Table 1).

**Table 1:** Microsatellite analysis of *Plasmodium vivax* infection strains

Sample	Date	Microsatellite markers (Chromosome position, marker ID)					
		3.35, v33	4.2771, v28	6.3, v30	7.67, v25	12.335, v24	14.185, v23
Initial infection	Feb-11	118	87	157	101	162	270
Second recurrence	Aug-11	118	87	157	101	162	270

The periodicity of the symptoms, the patient’s low risk of exposure, and the homology of the molecular data suggest that his second and third episodes of malaria were a relapse of the initial infection rather than a re-infection with a new parasite.

## Case discussion

Primaquine is currently the only FDA approved drug to prevent relapses of *P. vivax*, as it targets the hypnozoites latent in hepatic cells [1, 2]. Many Ministries of Health in South America recommend a maximum of 30 mg/day for 7 days (210 mg total) establishing a maximum weight of 60 kg and only a week of treatment as compliance is low for longer time periods [3]. It has been suggested, however, that this total dose may be at the lower edge of reasonable efficacy and minimally effective [4]. Several studies have demonstrated failure of primaquine due to inadequate dosage compared to body weight [1, 5–7]. In this case, the maximum total dose of 210 mg recommended by the Peruvian Ministry of Health [3] was not effective for this patient. The subject weighed 25 % over the 60 kg weight limit and therefore received low-dose regimens twice. No more relapses were observed after a 14-day full dosage treatment. This is of particular importance for the military, as personnel tend to weigh more than the weight (60 kg) used to calculate the maximum total dosage of primaquine. Additionally, the rise of obesity in malaria endemic countries could render standard treatment recommendations ineffective. Higher doses of primaquine are used in other regions, as current Centers for Disease Control and Prevention (CDC) standards advise a maximum dosage of 0.5 mg/kg/day capped at 60 kg (max of 30 mg/day) for no more than 14 days (420 mg total) in G6PD positive individuals [1]. However, compliance remains a critical issue. Tafenoquine, a new drug undergoing clinical evaluation, has been shown to be significantly effective in a single dose taken with chloroquine in preventing relapse of *vivax* malaria [8]. Until the safety and efficacy of tafenoquine can be further determined, the adoption of CDC recommendations and larger doses of primaquine are the only options to effectively treat patients and prevent relapse as well as contribute to the elimination of the parasite in endemic areas.

A key question in this case is whether the patient’s subsequent episodes of malaria were the result of relapse of dormant hypnozoites or re-infection with a new strain. After the initial hospitalization, the patient did not travel to any areas with malaria transmission, making re-infection unlikely. Additionally, the strains from the February and August blood smears were identical based on molecular analysis. The Amazon Basin is a region of high *P. vivax* diversity, and homologous strains would strongly suggest relapse [9]. While whole genomic analysis is necessary in order to completely determine if the strains are identical and therefore exhibiting relapse [10], re-infection is doubtful due to the low possibility of transmission, homologous strains between hospitalizations, and high genetic diversity among *P. vivax* species in this region. *P. vivax* exhibits geographical variation in relapse patterns [11]. Historically, it has been thought that frequent relapsing strains originate from the tropics, and that strains with a long latent period originate from temperate regions [12]. However, the Amazon Basin is unique in that it contains both long-latency and frequent relapsing strains, complicating attempts to characterize the predominant relapse pattern [11, 13]. Recent evidence has demonstrated that the majority of relapses occur within 2–3 months of infection, with 70 % of relapses occurring within 5 months of initial infection [11, 14–16]. In addition, characterizing relapse patterns in malaria-endemic areas is inherently problematic as studies cannot completely distinguish a relapse from a new infection. True periodicity of relapse can most accurately be determined in patients with single, short exposure that minimizes the chance of re-infection. Travellers, including the military, are therefore good populations to study relapse patterns. In this case, the sailor did not travel to any areas where he could have been re-infected. His recurrent parasitaemia is most likely due to the reactivation of a latent strain. The two relapses occurring 3 months apart are most likely representative of the relapse pattern common in the Amazon Basin.

In conclusion, the frequent incidence of relapse of *P. vivax* in South America poses an important challenge for elimination. The 60 kg cap on patient weight and 210 mg maximum primaquine dose recommended in Peru and other South American nations may limit the efficacy necessary for radical cure in individual patients and control in the endemic population. This becomes increasingly important during the current epidemic of obesity, as a heavier patient population could negatively impact the management of *vivax* malaria and potentially other diseases, as well as the effectiveness of other anti-malarial drugs. Until more effective radical cure drugs are licensed [8] or alternative parameters for dosing are explored (e.g. body mass index), clinicians can better prevent relapses in *vivax* malaria and support malaria control by using adequate primaquine dosing by weight and increasing the number of days of treatment up to 14 days if needed.
